# Association of obstructive sleep apnea with cardiometabolic diseases and cardiovascular mortality

**DOI:** 10.1111/crj.13666

**Published:** 2023-07-21

**Authors:** Jia Gao, Licheng Shi, Xuanfeng Zhu, Jiannan Liu

**Affiliations:** ^1^ Department of Respiratory Medicine Jiangsu Province Official Hospital Nanjing China

**Keywords:** cardiometabolic diseases, cardiovascular diseases, diabetes, hypertension, obstructive sleep apnea

## Abstract

**Background:**

Obstructive sleep apnea (OSA) is one of the leading respiratory disorders, increasing the risk of cardiometabolic diseases. In the study, we investigated the association between OSA and the risk of cardiometabolic diseases and all‐cause and cardiovascular mortality in adults.

**Methods:**

Participants were enrolled in the National Health and Nutrition Examination Survey. The baseline covariates were compared between participants with and without OSA status. Multivariable logistic regression was performed to explore the association between OSA and cardiometabolic diseases, while Cox proportional regression was performed for all‐cause and cardiovascular mortality.

**Results:**

OSA status was positively associated with higher risks of cardiometabolic diseases, including hypertension (odds ratio [OR] 1.28, 95% confidence interval [CI] 1.14–1.45; *p* < 0.001), diabetes (OR 1.46, 95% CI 1.22–1.76; *p* < 0.001), and cardiovascular diseases (OR 1.29, 95% CI 1.08–1.54; *p* = 0.006) after adjusting for numerous covariates. However, no associations of OSA with all‐cause or cardiovascular mortality were observed.

**Conclusion:**

OSA was associated with a higher risk of hypertension, diabetes, and cardiovascular diseases, but had no significant association with all‐cause or cardiovascular mortality in adults.

## Graphical abstract

The flow chart of participant selection.

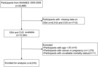

**Abbreviations**
AHI: apnea–hypopnea index
BMI: body mass index
CI: confidence interval
eGFR: estimated glomerular filtration rate
HDL‐C: high‐density lipoprotein cholesterol
HIFs: hypoxia‐inducible factors
LDL‐C: low‐density lipoprotein cholesterol
NCHS: National Center of Health Statistics
NHANES: National Health and Nutrition Examination Survey
OR: odds ratio
OSA: obstructive sleep apnea
PIR: poverty income ratio

## 1 INTRODUCTION

Obstructive sleep apnea (OSA) is one of the sleep‐related breathing disorders characterized by repetitive episodes of upper airway obstruction, nocturnal hypoxemia and hypercapnia, arousals, sympathetic stimulation, and exaggerated intrathoracic pressure swings.^1^ The apnea–hypopnea index (AHI) is the most commonly used metric to determine the diagnosis and severity classification of OSA,^2^ as well as the associations between OSA and comorbidities.^3^ According to an epidemiological review by Franklin and Lindberg, the prevalence of OSA was estimated to be 22% in men and 17% in women.^4^

It is well‐known that OSA is a cause of hypertension.^5^ Several studies have emphasized that OSA is an independent risk factor for coronary heart disease,^6^, ^7^ arrhythmia,^8^, ^9^ and stroke.^10^, ^11^ Metabolic disorders, including diabetes and impaired lipid metabolism, are also associated with OSA.^12^, ^13^ Additionally, more and more evidence has shown that OSA, independent of hypertension, could induce heart failure^14^ and other life‐threatening incidents.^15^ Therefore, OSA may be a detrimental co‐morbidity that severely impacts all‐cause and cardiovascular mortality.

Previous studies found that OSA was reported to be associated with all‐cause and cardiovascular mortality in patients with chronic obstructive pulmonary disease,^16^ acute coronary syndrome,^17^ and chronic kidney disease.^18^ However, data on the link between OSA and mortality in the general population was limited. In the present study, we aimed to examine the association between OSA and all‐cause and cardiovascular mortality in general adults. It will improve our insights into the prevention and management of OSA.

## 2 METHODS

### 2.1 Study population

We made a secondary analysis based on the National Health and Nutrition Examination Survey (NHANES) 2005–2008, a nationwide multistage‐sampling survey. Firstly, participants with missing data on OSA (*n* = 8310) and cardiometabolic diseases (*n* = 1715) were excluded. Participants with cancer and pregnancy (*n* = 1279) and unavailable mortality status (*n* = 11) were also excluded because pregnancy and cancer could have a cofounding effect on sleep status and mortality, respectively. A total of 9076 respondents were analyzed in this study. The study was approved by the Review Board of National Center of Health Statistics (protocol #2005‐06), and the written consent was obtained from the participants.

### 2.2 Exposure and outcomes

The presence of OSA was determined through a questionnaire that was administered by trained interviewers. Subjects who answered yes to the question “Have you ever been told by a doctor or other health professional that you have a sleep disorder?” and reported sleep apnea to “What was the sleep disorder?” were defined as having OSA.^19^

Cardiovascular diseases consisting of congestive heart failure, coronary heart disease, angina pectoris, heart attack, and stroke were obtained from the self‐reports. The questions included “Has a doctor or other health professional ever told you that you have congestive heart failure/coronary heart disease/angina pectoris/heart attack/stroke?”. Hypertension was diagnosed as previous hypertension, systolic blood pressure ≥140 mmHg, diastolic blood pressure ≥90 mmHg, or taking antihypertensive drugs. Diabetes was diagnosed as previous diabetes, fasting glucose >7 mmol/L, HBA1c >6.5%, or use of hypoglycemic drugs.

Mortality status was determined from the National Death Index by 31 December 2015. The ICD‐10 codes for cardiovascular diseases included I00–I09, I11, I13, I20–I51, I60–I69, and I70–I78.

### 2.3 Survey and measurement

A standard questionnaire was used to collect demographic information, including age, sex, race, educational level, poverty income ratio (PIR), body mass index (BMI), drinking and smoking status, history of diseases (hypertension, diabetes, and cardiovascular disease), and history of medication (antihypertensive drug, hypoglycemic drug, and lipid‐lowering drug). Smoking was categorized into current, former, and never. Race/ethnicity was divided into four groups: non‐Hispanic White or Black, Mexican American, and others. Education level included four categories: less than or equivalent to high school, college, or above. Height and weight were measured by physical examinations, and BMI was calculated as body weight divided by height squared. Serum levels of cholesterol, triglycerides, high‐density lipoprotein cholesterol (HDL‐C), and low‐density lipoprotein cholesterol (LDL‐C) were measured using an enzymatic assay. The estimated glomerular filtration rate (eGFR) was calculated based on the chronic kidney disease epidemiology collaboration (CKD‐EPI) equation.

### 2.4 Statistical analysis

Numeric parameters were expressed as the mean ± standard deviation, and categorical variables were presented as numbers (percentages). Differences between groups were compared using the analysis of variance for quantitative values and the chi‐squared test for qualitative parameters. A multivariable logistic regression analysis was used to explore the cross‐sectional relationship between OSA and cardiometabolic diseases. A Cox proportional regression was performed to explore the prognostic effect of OSA on all‐cause and cardiovascular mortality. Model 1 was unadjusted; Model 2 was adjusted for gender and age. Model 3 was adjusted for Model 2 covariates plus race, education, PIR, BMI, drinking, smoking, and activity. Model 4 was adjusted for Model 3 covariates plus medication use and cholesterol, triglycerides, HDL, LDL, and eGFR. All statistical analysis was performed using IBM SPSS 25.0. A *p* value < 0.05 was deemed statistically significant.

## 3 RESULTS

A total of 9076 individuals were included in our study. Compared with participants without OSA, the population with OSA tends to be older and male and has a higher percentage of diabetes, hypertension, and cardiovascular disease (Table 1).

**TABLE 1:** Baseline variables of the study participants according to OSA status.

Variables	No OSA (*n* = 4648)	OSA (*n* = 4428)	*p*
Male	1915 (43.2)	2725 (58.6)	<0.001
Age, years	56.84 (9.09)	59.99 (6.26)	<0.001
Race (%)			
Non‐Hispanic White	2042 (46.1)	2130 (45.8)	0.416
Non‐Hispanic Black	1009 (22.8)	1028 (22.1)	
Mexican American	857 (19.4)	892 (19.2)	
Others	520 (11.7)	598 (12.9)	
Education (%)			
Less than high school	1293 (29.2)	1398 (30.1)	0.002
High school or equivalent	1018 (23.0)	1188 (25.6)	
College or above	2111 (47.7)	2057 (44.3)	
PIR (%)			
<1	849 (20.8)	780 (17.9)	0.003
1–3	1714 (42.0)	1850 (42.6)	
>3	1521 (37.2)	1716 (39.5)	
BMI, kg/m^2^	27.1 (5.7)	30.6 (7.3)	<0.001
Drinking (%)	578 (48.4)	632 (57.0)	<0.001
Smoking (%)			
Current	773 (22.2)	987 (28.8)	<0.001
Past	168 (4.8)	182 (5.3)	
Never	2543 (73.0)	2257 (65.9)	
Activity (%)			
Vigorous	1166 (44.2)	1182 (43.3)	0.52
Moderate	1089 (41.3)	1168 (42.8)	
Inactive	381 (14.5)	378 (13.9)	
Past history (%)			
HBP	658 (16.6)	955 (22.3)	<0.001
DM	497 (11.2)	884 (19.0)	<0.001
CVD	288 (6.5)	450 (9.7)	<0.001
Prior medication (%)			
Antihypertensive drugs	986 (85.2)	1536 (86.5)	0.343
Hypoglycemic drugs	292 (53.1)	507 (56.1)	0.29
Lipid‐lowering drugs	541 (80.6)	849 (81.1)	0.861
Cholesterol, mg/dL	196.95 (40.72)	199.09 (42.78)	0.021
Triglycerides, mg/dL	144.45 (122.20)	173.82 (148.55)	<0.001
HDL, mg/dL	55.11 (16.11)	50.33 (15.56)	<0.001
LDL, mg/dL	113.00 (34.81)	116.14 (35.90)	0.006
eGFR, mL/min per 1.73 m^2^	91.05 (25.24)	89.49 (24.42)	0.005

*Note*: Data are presented as mean (SD) or *n* (%).

Abbreviations: BMI, body mass index; CVD, cardiovascular disease; DM, diabetes mellitus; eGFR, estimated glomerular filtration rate; HBP, high blood pressure; HDL, high‐density lipoprotein; LDL, low‐density lipoprotein; OSA, obstructive sleep apnea; PIR, poverty income ratio.

To investigate the association between OSA and cardiometabolic diseases (hypertension, diabetes, and cardiovascular diseases), we established multiple logistic regression models, as shown in Table 2. OSA was positively associated with higher odds of hypertension (OR = 1.44; 95% CI, 1.29, 1.60; *p* < 0.001), diabetes (OR = 1.86; 95% CI, 1.65, 2.09; *p* < 0.001), and cardiovascular diseases (OR = 1.54; 95% CI, 1.32, 1.80; *p* < 0.001) in Model 1. This association was not altered after adjusting for gender, age, race, education, PIR, BMI, drinking, smoking, activity, hypertension, diabetes, CVD, antihypertensive drugs, hypoglycemic drugs, lipid‐lowering drugs, cholesterol, triglycerides, HDL, LDL, and eGFR in Model 4. Furthermore, we investigated the association between OSA and all‐cause and cardiovascular mortality. Cox proportional regression analysis found that OSA had no significant association with all‐cause and cardiovascular mortality in all models (Table 3).

**TABLE 2:** Association of obstructive sleep apnea (OSA) with cardiometabolic diseases.

Cardiometabolic diseases	OR	95% CI	*p*
Hypertension			
Model 1	1.44	[1.29, 1.60]	<0.001
Model 2	1.37	[1.22, 1.54]	<0.001
Model 3	1.30	[1.15, 1.46]	<0.001
Model 4	1.28	[1.14, 1.45]	<0.001
Diabetes			
Model 1	1.86	[1.65, 2.09]	<0.001
Model 2	1.85	[1.63, 2.09]	<0.001
Model 3	1.37	[1.20, 1.57]	<0.001
Model4	1.46	[1.22, 1.76]	<0.001
Cardiovascular diseases			
Model 1	1.54	[1.32, 1.80]	<0.001
Model 2	1.51	[1.28, 1.79]	<0.001
Model 3	1.36	[1.14, 1.62]	0.001
Model 4	1.29	[1.08, 1.54]	0.006

*Note*: Model 1: unadjusted. Model 2: adjusted for gender and age. Model 3: adjusted for gender, age, race, education, PIR, BMI, drinking, smoking, and activity. Model 4: adjusted for gender, age, race, education, PIR, BMI, drinking, smoking, activity, antihypertensive drugs, hypoglycemic drugs, lipid‐lowering drugs, cholesterol, triglycerides, HDL, LDL, and eGFR.

Abbreviations: BMI, body mass index; CI, confidence interval; eGFR, estimated glomerular filtration rate; HDL, high‐density lipoprotein; LDL, low‐density lipoprotein; OR, odds ratio; PIR, poverty income ratio.

**TABLE 3:** Association of obstructive sleep apnea (OSA) with all‐cause and cardiovascular mortality.

	HR	95% CI	*p*
All‐cause mortality			
Model 1	0.93	[0.82, 1.04]	0.206
Model 2	0.97	[0.86, 1.10]	0.634
Model 3	0.99	[0.87, 1.12]	0.886
Model 4	0.96	[0.84, 1.09]	0.507
Cardiovascular mortality			
Model 1	0.96	[0.72, 1.28]	0.787
Model 2	0.94	[0.70, 1.25]	0.658
Model 3	0.94	[0.70, 1.27]	0.685
Model 4	0.91	[0.67, 1.23]	0.532

*Note*: Model 1: unadjusted. Model 2: adjusted for gender and age. Model 3: adjusted for gender, age, race, education, PIR, BMI, drinking, smoking, and activity. Model 4: adjusted for gender, age, race, education, PIR, BMI, drinking, smoking, activity, antihypertensive drugs, hypoglycemic drugs, lipid‐lowering drugs, cholesterol, triglycerides, HDL, LDL, and eGFR.

Abbreviations: BMI, body mass index; CI, confidence interval; eGFR, estimated glomerular filtration rate; HDL, high‐density lipoprotein; HR, hazard ratio; LDL, low‐density lipoprotein; PIR, poverty income ratio.

## 4 DISCUSSION

Our study concluded that OSA was associated with the presence of cardiometabolic diseases, including diabetes, hypertension, and cardiovascular diseases, but had no significant relationship with all‐cause or cardiovascular mortality.

Punjabi et al. showed that severe hypopneas were independently associated with cardiovascular diseases.^20^ Appleton et al. conducted a longitudinal study showing that hypertension was related to OSA in community‐dwelling men.^21^ The prevalence of OSA in people with type 2 diabetes mellitus was higher than the general population.^22^ Besides, OSA was associated with atherosclerosis^23^ and coronary plaque,^24^ which all laid the foundations for increased risk of cardiovascular diseases. Our studies confirmed these associations among a survey‐based general population. Increased sympathetic nervous system activity, oxidative stress, and systemic inflammation^25^, ^26^, ^27^ may be the mechanisms underlying the association between OSA and cardiometabolic diseases. Another important factor was hypoxia‐inducible factors (HIFs).^28^ Intermittent hypoxia increased the expression of HIFs, which activated the burst of reactive oxygen species (ROS). ROS, in turn, stimulates the sympathetic nervous system and insulin resistance.^29^

Recent study showed that hypoxemic burden because of sleep apnea was strongly predictive of cardiovascular diseases related mortality.^30^ Lavie et al. conducted a large prospective cohort study and concluded that severe OSA increased the risk of all‐cause mortality in middle‐aged male patients.^31^ Marin et al. also concluded that those with severe OSA were at higher risk for both fatal and non‐fatal cardiovascular events.^5^ Besides, a multitude of studies reported that OSA increased the all‐cause mortality of patients with lung cancer,^32^ asthmatics,^33^ and metabolic syndrome.^34^ However, using a nationwidely general population enrolled in NHANES, no associations between OSA and all‐cause and cardiovascular mortality was observed. The difference could be because OSA diagnosis was based on self‐reports rather than objective tests and lack of severity evaluation. The general population enrolled in our study may have a higher proportion of mild OSA, and mild hypoxia may be protective against cardiovascular mortality. Besides, short‐duration and low‐intensity intermittent hypoxia may provide a degree of protection against ischemic events, which may explain no predictive value in cardiovascular mortality,^35^ which may weaken the associations. The underlying reasons need further investigation.

Some limitations existed in our study. First, the main limitation of this study is the lack of an objective assessment of OSA. Some data, including the diagnosis of OSA and cardiovascular diseases, were obtained from self‐reported interviews. This may have overestimated the true burden of OSA. Second, even though we excluded individuals with cancer and pregnancy, other chronic conditions, like COPD, autoimmune disease, and chronic kidney disease, were not all excluded, which are linked to worse outcomes and poorer survival. Finally, there is a lack of knowledge regarding the treatment of OSA or not, such as adequate control or good compliance with CPAP, which has an important effect on the mortality in OSA.^36^, ^37^ Further research should evaluate the association across the subgroup with or without CPAP treatment.

## 5 CONCLUSION

In conclusion, we found that OSA was associated with the presence of cardiometabolic diseases, including diabetes, hypertension, and cardiovascular diseases. However, it could not predict mortality in the general population.

## AUTHOR CONTRIBUTIONS

Xuanfeng Zhu and Jiannan Liu designed the study. Jia Gao wrote the manuscript. Licheng Shi performed the statistical analysis. All authors approved the final manuscript.

## CONFLICT OF INTEREST STATEMENT

The authors have no conflicts of interest.

## ETHICS APPROVAL

The study was approved by the Review Board of National Center of Health Statistics (protocol #2005‐06) and the written consent was obtained from the participants.

## Data Availability

All data could be obtained upon request from Xuanfeng Zhu.
